# Risk Factors beyond Chemotherapy Exposure for Secondary Myeloid Neoplasms after Hematologic Cancers: A SEER-Based Study

**DOI:** 10.1158/2767-9764.CRC-25-0340

**Published:** 2025-12-11

**Authors:** Abhay Singh, Theresa Hahn, Rahul Mishra, Megan M. Herr, Swapna Thota

**Affiliations:** 1 Department of Hematology Oncology, Taussig Cancer Institute, Cleveland Clinic, Cleveland, Ohio.; 2 Department of Cancer Prevention and Control, Roswell Park Comprehensive Cancer Center, Buffalo, New York.; 3 Department of Internal Medicine, Anne Arundel Medical Center, Annapolis, Maryland.; 4 Department of Medicine, Roswell Park Comprehensive Cancer Center, Buffalo, New York.; 5 Division of Hematology/Oncology, University of Tennessee Health Science Center, Memphis, Tennessee.

## Abstract

As survival rates for lymphoid malignancies continue to improve, understanding the late effects after treatment, such as secondary myeloid neoplasms (sMN), is increasingly critical for survivorship. This large-scale population-based (SEER-Medicare) study investigated the risk factors and cumulative incidence of sMNs in patients with diagnosis of diffuse large B-cell lymphoma (DLBCL), follicular lymphoma (FL), and multiple myeloma between 2000 and 2011, with follow-up through 2015. Patients with FL had a continuous increase in the cumulative incidence of sMNs with longer follow-up, whereas incidence of patients with multiple myeloma plateaued after 60 months. Notably, patients with DLBCL diagnosed in more recent years (2004–2007 and 2008–2011) had a higher cumulative sMN incidence compared with those diagnosed earlier (2000–2003). In the Medicare population, older age at diagnosis was associated with significant increase in sMN risk in patients with DLBCL and FL. Chemotherapy exposure or G-CSF exposure significantly increased sMN risk across all three malignancies. Chronic autoimmune conditions increased sMN risk in patients with DLBCL. These findings provide crucial insights into sMN risk factors. Chemotherapy exposure is a recognized risk factor, and comorbidities such as a history of autoimmunity and G-CSF exposures have been identified as additional mediators of sMN risk in our study.

**Significance:**

Our study reveals population-level risk factors for sMNs, including novel links to autoimmune disease and G-CSF, in addition to known causes like chemotherapy/radiation. These findings underscore the complex pathogenesis of sMNs and the need for molecular data in prospective studies to guide prevention, detection, and survivorship care.

## Introduction

Myelodysplastic syndromes (MDS) and acute myeloid leukemia (AML) occur as late complications of cytotoxic treatments for primary malignancies, including lymphoid neoplasms (1). The risk of developing these myeloid neoplasms (MN) is especially elevated in patients with lymphoid malignancies such as diffuse large B-cell lymphoma (DLBCL), follicular lymphoma (FL), and plasma cell myeloma (multiple myeloma) due to the intensive chemotherapy and radiation treatments used in their management (2, 3). The pathogenesis of these secondary MNs (sMN) is complex, involving the interplay of treatment-related factors, patient characteristics, and underlying genetic predispositions. Conventional chemotherapeutic agents, particularly alkylating agents and topoisomerase II inhibitors, have long been recognized as primary contributors to therapy-related MN development (2, 4). However, the evolving landscape of cancer therapeutics, with widespread adoption of targeted therapies and immunomodulators, we reevaluated risk factors and incidence patterns of sMNs. Recent studies have highlighted the potential role of clonal hematopoiesis (CH) in the development of MNs (5–7). CH, characterized by the presence of somatic mutations in hematopoietic stem cells, is increasingly prevalent with age and may be exacerbated by cytotoxic therapies (8, 9). The expansion of these preexisting mutant clones under the selective pressure of chemotherapy plays a key role in sMN pathogenesis (10–12). Furthermore, supportive care measures, particularly G-CSF, have become standard practice in the management of chemotherapy-induced neutropenia. This lowered the risk of infectious complications but linked with increased risk of sMN development (13–16). Similarly, sMNs have also been associated to autoimmune diseases, though it remains unclear whether this association is driven by underlying immune dysregulation or the use of immunosuppressive therapies (17–19).

As survival rates for lymphoid malignancies continue to improve, understanding the late effects after treatment is critical for survivorship. In our recent study of the five common types of solid cancers (breast, prostate, gastrointestinal, lung, and bladder), we noted history of autoimmune disease and G-CSF exposure emerged as consistent predictors of sMNs, in addition to the known risk factors of chemotherapy and radiation exposure (20). In this study, we aim to investigate the risk factors and cumulative incidence of sMNs among patients with non-Hodgkin lymphomas (DLBCL and FL) as well as multiple myeloma using data from the large population-based Surveillance, Epidemiology, and End Results (SEER)-Medicare claims registry.

## Materials and Methods

### Population

We conducted a population-based retrospective cohort study using the SEER-Medicare linked database (RRID: SCR_025811). Medicare claims data were used to identify medical conditions from hospitals, physician offices, and outpatient clinics. For patients more than 65 years of age, 97% are eligible for Medicare. Almost all have part A coverage, which includes hospital, skilled nursing facility, hospice, and some home health care claims. Of these patients, 96% enrolled in part B which covers physician and outpatient services. This study was conducted under a data use agreement with NIH–SEER, using deidentified data. In accordance with the Declaration of Helsinki and institutional guidelines, it was exempt from informed consent requirements.

A total of 39,601 adults diagnosed with a first primary lymphoid hematologic neoplasm (DLBCL, FL, or multiple myeloma) between 2000 and 2011, with follow-up through 2015, were selected from the SEER cancer file (previously named as Patient Entitlement and Diagnosis Summary File). A 1-year latency period between their primary cancer and sMN diagnoses (Supplementary Table S1) was required to avoid surveillance bias and 1-year minimum follow-up was required of all patients. Age at first cancer diagnosis was limited to 66 to 85 years to account for decreased surveillance of individuals more than 85 years. Individuals who were diagnosed at autopsy or by death certificate were excluded. Medical conditions identified *a priori* were evaluated using data from Medicare claims. They were included in the analysis if they occurred at least 2 months prior to sMN diagnosis but could have occurred before or after their first primary diagnosis. SEER files were used to identify exposure to chemotherapy or radiotherapy. Medical conditions and covariates in model construction included age, gender, race, prior receipt of chemotherapy and radiotherapy, history of hematopoietic stem cell transplant, prior history of cardiovascular disease (stroke, heart disease, and other vascular diseases), history of autoimmune disease, hypertension, diabetes, infections, and exposure to G-CSF (often used interchangeably with the term “growth factors”; see Supplementary Table S2 for different covariate definitions). Autoimmune diseases were categorized as acute (conditions typically requiring short course of immunosuppressive therapy) or chronic (requiring continuous maintenance immunosuppressive therapy for a prolonged period over multiple years; Supplementary Table S3). Race and ethnicity were categorized as White, Black, and other/unknown. Individuals were classified as having a condition of interest if they had at least one hospital claim or two physician/outpatient claims at least 30 days apart. To reduce the risk of bias resulting from differential exposure assessment, in which patients closer to a sMN diagnosis may undergo more frequent and detailed medical evaluations, potentially inflating the detection of exposures, we excluded any medical conditions or treatments documented within 2 months prior to the sMN diagnosis. Patients were excluded if diagnosis month was unknown.

### Statistical analyses

Descriptive analyses were conducted to compare potential risk factors for sMNs including infections, autoimmune diseases, and cardiovascular conditions. Associations between these factors and sMNs were assessed using *χ*^2^ tests. Variables significantly associated with sMNs in univariate analyses were included in the multivariable model. Analyses were also adjusted for latency, duration of part A, part B non-health maintenance organization coverage, and number of physician visits, hospital, or outpatient claims, if associated with sMN. Cox proportional hazards model was assessed for fit accounting for the competing risk of death, and statistical significance was set at *P* < 0.01 to control for multiple comparisons. Models were stratified by the type of first primary malignancy. Cumulative incidence curves of time to sMN diagnosis by first primary cancer were calculated and stratified by year of first primary cancer diagnosis accounting for the competing risk of death. Data were analyzed using SAS v9.4 (RRID: SCR_008567).

## Results

We identified a total of 39,601 patients diagnosed with DLBCL (*n* = 14,584), FL (*n* = 10,115), and multiple myeloma (*n* = 14,902) per inclusion criteria. Among them, 372 developed MNs, including MDS or AML: 157 in DLBCL, 117 in FL, and 98 in multiple myeloma.

### Patient characteristics

Women (51%) and White race comprised 85% of the study population. Whereas the majority of patients were White, among patients with multiple myeloma, there was a high representation of Black patients (17%). A higher proportion of sMN cases had a history of chemotherapy, G-CSF, and chronic autoimmune conditions compared with those without sMNs.

In DLBCL, 110/157 (70.1%) of patients with sMNs received chemotherapy versus 6,885/14,427 (47.7%) without sMNs. In FL, 79/117 (67.5%) of patients with sMNs received chemotherapy compared with 3,848/9,998 (38.5%) without sMNs, and in multiple myeloma, 56/98 (57.1%) of those with sMNs received chemotherapy versus 5,217/14,804 (35.2%) without sMNs. The remaining proportions were categorized as “no/unknown,” reflecting either lack of treatment or incomplete treatment documentation (Table 1). Of note, 8% of the population had a history of autoimmune disease, and 12% had exposure to G-CSF. G-CSF use was also more common among patients who developed sMNs across all cancer types.

**Table 1.** Characteristics of patients with and without sMNs after index diagnosis of DLBCL, FL, or multiple myeloma diagnosed 2000–2011 using SEER-Medicare, expressed in numbers.

	DLBCL		FL		MM	
	sMN *n* = 157	No sMN *n* = 14,427	sMN *n* = 117	No sMN *n* = 9,998	sMN *n* = 98	No sMN *n* = 14,804
Age at first primary cancer						
<70 years	47	3,393	36	2,690	30	3,619
70 – <75 years	47	4,147	31	2,968	30	4,408
≥75 years	63	6,887	50	4,340	38	6,777
Median (range), years	72 (66–84)	74 (66–84)	73 (66–84)	73 (66–84)	73 (66–83)	74 (66–84)
Sex						
Male	75	7,017	63	4,513	58	7,647
Female	82	7,410	54	5,485	40	7,157
Race						
White	>146	12,810	>106	9,221	>71	11,465
Black	<11	537	<11	344	16	2,497
Other/unknown	<11	1,080	<11	433	<11	842
Year of first primary cancer diagnosis						
2000–2003	45	4,527	55	3,142	31	4,481
2004–2007	46	4,747	34	3,436	33	4,682
2008–2011	52	5,153	28	3,420	34	5,641
Initial chemotherapy						
No/unknown	47	7,542	38	6,150	42	9,587
Yes	110	6,885	79	3,848	56	5,217
Initial radiation						
No/unknown radiation	121	11,560	87	8,317	85	12,892
Radiation	36	2,867	30	1,681	13	1,912
Autologous or allogeneic HCT						
No HCT	141	12,993	>106	9,086	81	12,837
HCT	16	1,434	<11	912	17	1,967
Cardiovascular disease						
No cardiovascular disease	31	2,350	25	1,770	17	2,405
Cardiovascular disease	126	12,077	92	8,228	81	12,399
Acute autoimmune conditions						
No autoimmune conditions	143	13,305	103	9,295	87	13,679
Autoimmune conditions	14	1,122	14	703	11	1,125
Chronic autoimmune conditions						
No autoimmune conditions	121	12,025	96	8,510	83	12,644
Autoimmune conditions	36	2,402	21	1,488	15	2,160
Infection						
No infection	63	5,078	40	3,843	34	4,299
Infection	94	9,349	77	6,155	64	10,505
G-CSF						
No G-CSF	82	11,657	64	8,768	72	14,041
G-CSF	75	2,770	53	1,230	26	763
Duration of Medicare coverage						
2 to <139 months	71	2,336	47	1,468	>35	4,167
139 to <190 months	39	3,412	29	2,331	26	4,554
190 to <240 months	26	3,855	26	2,793	26	3,638
≥240 months	21	4,824	15	3,406	<11	2,445

DLBCL, ICD-O-3: 9680, 9684, 9688, 9735, and 9738; FL, ICD-O-3: 9690-9691, 9695, and 9698; MM, ICD-O-3: 9732.

Abbreviations: HCT, hematopoietic stem cell transplantation; ICD, International Classification of Diseases; MM, multiple myeloma.

### Cumulative incidence of sMNs

**Figure 1.**
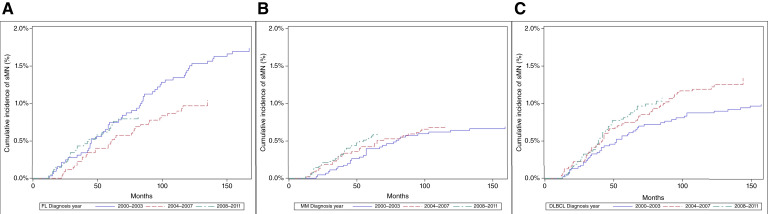
The cumulative incidence of sMNs by diagnosis year cohorts: 2000–2003 (blue line), 2004–2007 (red dashed line), and 2008–2011 (green dashed line) in patients with (**A**) FL, (**B**) multiple myeloma (MM), and (**C**) DLBCL.

Kaplan–Meier analyses (Fig. 1A–C) showed that the cumulative incidence of sMNs varied by the year of first cancer diagnosis. The cumulative incidence of sMNs was assessed after DLBCL, multiple myeloma, and FL diagnoses across three time periods: 2000 to 2003, 2004 to 2007, and 2008 to 2011. In patients with FL, the cumulative incidence of sMNs continued to increase proportionally with longer follow-up, with no plateau in the curves out to 150 months. sMN incidence was the highest for patients diagnosed in the earliest cohort, 2000 to 2003, followed by 2008 to 2011 and then 2004 to 2007 (Fig. 1A). For patients with multiple myeloma, sMN incidence is the highest in the first 5 to 7 years after diagnosis, after which it plateaus; no significant difference is seen between time periods (Fig. 1B). Among patients with DLBCL, sMN incidence was higher in the two most recent time periods (2008–2011 and 2004–2007), compared with 2000 to 2003, suggesting a possible change in exposure leading to increased incidence after 2003 (Fig. 1C).

### sMN (secondary AML/MDS) risk factors

**Figure 2.**
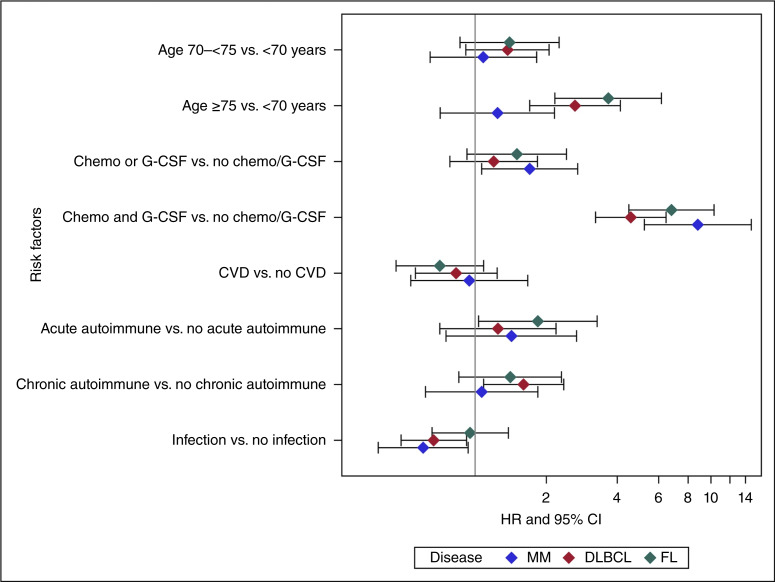
Forest plot depicting factors associated with increased risk of sMNs in patients with FL( green diamond), multiple myeloma (MM, blue diamond), and DLBCL (red diamond). Chemo, chemotherapy; CVD, cardiovascular disease.

Initial multivariable models considered the same set of risk factors in all three primary diseases (Fig. 2; Supplementary Table S4). As expected, older age (≥75 years) was significantly associated with an increased risk of sMNs in DLBCL and FL (*P* < 0.0001) but not patients with multiple myeloma. Exposure to chemotherapy and posttreatment G-CSF was significantly associated with an increased risk of sMN in DLBCL [HR, 4.56; 95% confidence interval (CI), 3.23–6.43; *P* < 0.0001], FL (HR, 6.79; 95% CI, 4.48–10.28; *P* < 0.0001), and multiple myeloma (HR, 8.77; 95% CI, 5.21–14.79; *P* < 0.0001). The history of chronic autoimmune disease significantly increased sMN risk, however, only in patients with DLBCL (DLBCL HR, 1.60; 95% CI, 1.09–2.37; *P* = 0.02; FL HR, 1.41; 95% CI, 0.85–2.32; *P* = 0.2; multiple myeloma HR, 1.07; 95% CI, 0.62–1.84; *P* = 0.8). Among other factors analyzed, history of infections was associated with reduced risk of sMN development in DLBCL (HR, 0.67; 95% CI, 0.49–0.92; *P* = 0.01) and multiple myeloma (HR, 0.60; 95% CI, 0.39–0.93; *P* = 0.02).

Given that risk factors differed by primary malignancy, separate multivariable models were constructed for each index disease. Significant risk factors for sMNs in patients with DLBCL included older age, history of chronic autoimmune disease, and chemotherapy and G-CSF usage, whereas infections were associated with lowered sMN risk (Supplementary Table S5). In patients with FL, older age, chemotherapy exposure, and G-CSF use were associated with an increased risk of sMNs (Supplementary Table S6). Patients with FL diagnosed in earlier years (2000–03), who had longer follow-up duration, also demonstrated an increased risk of developing sMNs. In patients with multiple myeloma, initial chemotherapy and G-CSF use (HR, 9.43; 95% CI, 5.75–15.47; *P* < 0.0001) was significantly associated with an increased risk of sMNs. In contrast, history of infections was associated with a decreased risk (HR, 0.59; 95% CI, 0.40–0.89; *P* = 0.01). Unlike in FL and DLBCL cohorts, older age was not a significant predictor of sMN risk in patients with multiple myeloma (Supplementary Table S7).

## Discussion

With the growing population of cancer survivors in the United States, the risk of sMNs has become an increasing concern. This large-scale analysis using a national population-based registry linked with claims data offers valuable insights into the incidence and risk factors of sMNs among patients with both indolent and aggressive lymphoid malignancies. Our findings reinforce known risk factors and identify novel predictors that may inform future risk of sMNs and shape survivorship care.

In addition to well-established traditional risk factors of sMN development such as older age and chemotherapy exposure, our findings point to potential roles for additional mediators or selection pressures that may influence CH and promote leukemic transformation. G-CSF exposure and autoimmunity may putatively be considered CH surrogates (7, 11). The inclusion of these CH surrogates in our analysis was unique and particularly important, as it allowed us to account for underlying biological processes that may predispose certain individuals to CH which ultimately may progress to sMNs. Recognizing these factors is crucial for improving risk stratification and understanding the broader context of sMN pathogenesis beyond cytotoxic treatment alone.

A key finding of our study is the association between G-CSF exposure and elevated sMN risk across all three lymphoid neoplasms examined. It remains unclear whether the increased risk stems from the dose-intense chemotherapy (which potentially include DNA-damaging agents) necessitating G-CSF use or from G-CSF exposure itself. G-CSF may also serve as a surrogate marker for more intensive chemotherapy regimens. In our previous research, we demonstrated that abrupt leukocytosis is associated with expansion of CH mutation (21). These observations make it plausible that G-CSF exposure leads to clonally expanded (CH-driven) leukocytosis by preferentially stimulating mutant clones, with potential implications for sMN risk. It is also plausible that use of G-CSF may be more common in patients experiencing prolonged hematologic recovery during chemotherapy, potentially reflecting an underlying deficit in hematopoietic reserve. Whereas these hypotheses remain speculative, they highlight the need for prospective studies incorporating molecular profiling to further explore the relationship between G-CSF exposure, hematopoietic reserve, and CH-driven clonal evolution (22).

Patients diagnosed with FL or DLBCL at older ages exhibited an elevated risk of sMN development. This finding aligns with the concept of accumulation of somatic mutations with advanced age (23). Previous studies have noted a high prevalence of CH in patients with DLBCL, which is associated with an elevated risk of developing sMNs (24). Several groups eloquently demonstrated that selective pressure from chemotherapy expands preexisting CH clones, leading to sMN development in non-Hodgkin lymphoma cohorts (5, 7, 25).

It was intriguing to observe the decreased risk of sMNs in association with infections. Although the exact mechanisms behind this observation remain unclear, one hypothesis suggests that recurrent infections skewed immune activity. Whether this leads to increased immune surveillance and early elimination of leukemic clones or alternatively, subtypes such as Epstein–Barr virus (EBV)-positive DLBCL has a poor prognosis compared with EBV-negative DLBCL, resulting in shorter survival for EBV-positive cases (26), thus reducing the likelihood of developing sMNs, a concept also referred to as immortal time bias (27).

Another notable finding from our study, previously reported in relation to myeloid malignancies such as MDS and chronic myelomonocytic leukemia, was the association between the history of autoimmune diseases and risk of sMNs (19, 28). The increased risk was particularly evident following DLBCL but not after FL or multiple myeloma, suggesting a potentially distinct immunopathologic contribution in DLBCL. These findings support the broader role of immune dysregulation in malignancies like DLBCL (29). Beyond the immunosuppressive effects of lymphoma itself, severe immune dysregulation associated with autoimmune diseases may also contribute to sMN pathogenesis, as supported by our population-level observation (30). Furthermore, we observed that 8% of our study population had a history of autoimmune disease, compared with a prevalence of less than 5% reported in the general population (31). This elevated prevalence further supports a potential link between autoimmunity and malignancy, reinforcing the hypothesis that autoimmune dysregulation may serve as a shared risk factor or co-driver of both lymphoid and myeloid neoplasms. On the other hand, widespread use of rituximab in DLBCL may portend B-cell aplasia or prolonged hypogammaglobulinemia. Our analysis is limited by the absence of treatment-specific details in the SEER database. The effect of such immunodeficiency on CH and sMNs should be explored in future studies.

When examining temporal trends, we observed a higher incidence rate of sMNs among patients diagnosed with DLBCL during more recent calendar periods (2004–2007 or 2008–2011) compared with 2000 to 2003. It is possible that improvement in survival with contemporary regimens could reduce the competing risk of death and thereby contribute to higher observed sMN incidence. This pattern also aligns with potential CH-favoring changes in DLBCL treatment, notably the widespread adoption of rituximab-based regimens and increased use of dose-dense therapies such as dose-adjusted EPOCH (2, 32), which often requires prophylactic G-CSF (33). For patients with multiple myeloma, we observed that the cumulative incidence of sMN reached a plateau after about 5 years. This plateau suggests a potential window of highest risk during the early years following diagnosis. Patients with multiple myeloma often receive the alkylating agent melphalan as part of their treatment, which is known to have a typical latency period of 5 to 7 years for the development of sMNs (34, 35). This latency period aligns with the findings in our study. Patients diagnosed with FL in the earlier period (2000–2003) exhibited a higher cumulative incidence of sMNs. This observation may be attributed to the extended follow-up duration for this group, a consequence of FL’s characteristically indolent nature and favorable overall survival rates (36). The prolonged survival of these patients potentially allows for an increased temporal window during which clonal evolution could occur, elevating the risk of sMN development (37).

Although our study provides valuable insights into sMN risk factors in patients with lymphoid neoplasms, we acknowledge limitations to its design and data source. Being retrospective in nature, this study introduces potential biases and limits our ability to establish causal relationships. A key limitation of our analysis is the low sensitivity of radiotherapy data within SEER. Due to the potential misclassification of treatment status, patients labeled as not having received radiation or chemotherapy may in fact have received treatment that was not captured in the registry. Thus, those with missing or incomplete records are grouped as “no/unknown.” Accordingly, treatment prevalence and comparisons by treatment status should be interpreted with caution in the context of our analysis. The inclusion of individuals without complete claims introduces the potential for misclassification bias as some exposures or diagnoses may not have been fully captured. However, any resulting misclassification would likely be nondifferential, meaning it would affect all groups similarly and therefore bias the observed associations toward the null, potentially dilute associations, underestimating the true effect sizes. Moreover, the absence of genomic data is a crucial limitation as genetic factors play a significant role in the development of sMNs. The population-based nature of our data source also means that we may have missed some cases of sMNs, particularly if patients sought treatment outside the captured healthcare system. Additionally, the accuracy of diagnosis coding in administrative databases can be variable, potentially leading to misclassification of some cases. Lastly, the impact of obesity and smoking could not be assessed due to inconsistency of the available data. Due to time-period constraints, this study does not account for risk of sMNs in the contemporary era of chimeric antigen receptor T-cell (CAR-T) therapy, which has drastically changed the treatment paradigm of lymphoma since its first approval in 2017. The recent studies evaluating CAR-T therapy have reported rapid evolution of existing CH, particularly TP53-mutated clones in patients with multiple myeloma, and expansion of CH and a shorter duration, compared with traditional chemotherapy, to sMNs in patients with LBCL (38–40). Cumulatively, these studies hypothesize multifactorial mechanism, involving CAR-T–related CH, cytokine-mediated prosurvival signaling in myeloid progenitors, and potential off-target effects of CAR-T cells on hematopoietic stem and progenitor cells fostering leukemogenic evolution (38–40). Despite our limitations, the large scale of our study and the consistency of our findings with previous research in other cancer types (41) lend credibility to our results.

Overall, our findings underscore the importance of continued research into the long-term effects of cancer therapies and the impact of comorbid conditions during survivorship. Future prospective studies, particularly those incorporating molecular analyses, may help clarify the relationship between G-CSF exposure and sMN development and inform potential risk mitigation strategies. The observed variations in sMN risk across calendar periods for patients with DLBCL also points to the importance of long-term survivorship monitoring, particularly as new therapies emerge.

### Conclusion

In conclusion, our study provides valuable population-level insights into the risk factors associated with sMNs. In addition to well-established contributors such as chemotherapy and radiation, we identified novel potential mediators, including autoimmune conditions and G-CSF exposure, that may influence sMN development. These findings highlight the complexity of sMN pathogenesis and the need for more detailed, prospective studies incorporating molecular analyses to better understand underlying mechanisms. Such efforts are essential to inform strategies for prevention, early detection, and long-term survivorship care.

## Supplementary Material

Supplemental Table S1 Definitions of sMDS/sAML by morphology and topography codes

Supplemental Table S2 ICD-9 and 10 Codes of Covariates

Supplemental Table S3 Classification of Autoimmune Conditions Based on Immunological Status and Duration of Immunosuppression

Supplemental Table S4 Risk of secondary MN after first primary DLBCL, FL, or MM diagnosed 2000-2011 using SEER-Medicare

Supplemental Table S5 Risk of sMN after first primary DLBCL diagnosed 2000-2011 using SEER-Medicare

Supplemental Table S6 Risk of sMN after first primary FL diagnosed 2000-2011 using SEER-Medicare

Supplemental Table S7 Risk of sMN after first primary MM diagnosed 2000-2011 using SEER-Medicare

## Data Availability

The data utilized in this study were obtained from the SEER-Medicare linked database after application and approval from the NCI SEER-Medicare. Access to these data is subject to restrictions and is available to researchers through application to the NCI (https://healthcaredelivery.cancer.gov/seermedicare). Due to the nature of the data agreements, the raw data cannot be made publicly available without prior NCI SEER-Medicare permission.
