# Supplementary material for: Risk Factors beyond Chemotherapy Exposure for Secondary Myeloid Neoplasms after Hematologic Cancers: A SEER-Based Study
Source: Cancer Res Commun. 2025 Dec 11;5(12):2149–56. doi: 10.1158/2767-9764.CRC-25-0340 (PMC12696405; doi:10.1158/2767-9764.CRC-25-0340)
Supplement: Supplemental Table S1 — Definitions of sMDS/sAML by morphology and topography codes [file crc-25-0340_supplemental_table_s1_suppst1.docx]

**Supplemental Table S1**: Definitions of sMDS/sAML by morphology and topography codes

| **Cancer Type** | **Morphology Codes** | **Topography Codes** |
| --- | --- | --- |
| sMDS | 9980, 9982-9983, 9985-9986, 9989, 9991-9992 | all topography codes |
| sAML | 9727, 9840, 9861, 9865-9867, 9869-9874, 9891, 9895-9898, 9910-9911, 9920, 9930-9931, 9984, 9987 | all topography codes |
| sMDS: secondary myelodysplastic syndromes; sAML: secondary acute myeloid leukemia | | |
