# Supplementary material for: Risk Factors beyond Chemotherapy Exposure for Secondary Myeloid Neoplasms after Hematologic Cancers: A SEER-Based Study
Source: Cancer Res Commun. 2025 Dec 11;5(12):2149–56. doi: 10.1158/2767-9764.CRC-25-0340 (PMC12696405; doi:10.1158/2767-9764.CRC-25-0340)
Supplement: Supplemental Table S2 — ICD-9 and 10 Codes of Covariates [file crc-25-0340_supplemental_table_s2_suppst2.docx]

**Supplemental Table S2**: ICD-9 and 10 Codes of Covariates

| **Condition** | **ICD-9 CM code** | **ICD-10 CM code** |
| --- | --- | --- |
| Hypertension | 7962, 4019, 40200, 40201, 40210, 40211, 40290, 40291 | R03.0, I10, I11.9, I11.0 |
| Heart disease | 41000-41002, 41010-41012 41020-41022, 41030-41032, 41040-41042, 41050-41052, 41060-41062, 41070-41072, 41080-41082, 41090-41092, 4110-4111, 41181, 41189, 412, 4130-4131, 4139, 41400-41407, 4142-4144, 4148-4149, 4299 | I21.09, I21.19, I21.11, I21.29, I21.4, I21.3, I24.1, I20.0, I24.0, I24.8, I25.2, I20.8, I20.1, I20.8, I25.10, I25.810, I25.811, I25.812, I25.82, I25.83, I25.84, I25.5, I25.9, I51.9 |
| Stroke | 43300-43301, 43310-43311, 43320-43321, 43330-43331, 43380-43381, 43390-43391, 43400-43401, 43410-43411, 43490-43491, 4350-4353, 4358-4359, 436, 4370, 4378-4379 | I65.1, I63.22, I65.29, I63.139, I65.09, I63.019, I65.8, I63.59, I65.8, I63.59, I65.9, I63.20, I66.09, I63.30, I66.09, I66.9, I63.50, G45.0, G45.8, G45.1, G45.9, I67.89, I67.2, I67.89, I67.9 |
| Atherosclerotic arterial disease | 4400-4401, 44020-44024, 44029-44032, 4404, 4408-4409 | I70.0, I70.1, I70.209, I70.219, I70.229, I70.25, I70.269, I70.299, I70.399, I70.499, I70.599, I70.92, I70.8, I70.90 |
| Diabetes | 25001, 25003, 25011, 25013, 25021, 25023, 25031, 25033, 25041, 25043, 25051, 25053, 25061, 25063, 25071, 25073, 25081, 25083, 25091, 25093, 24900-24901, 24910-24911, 24920-24921, 24930-24931, 24940-24941, 24950-24951, 24960-24961, 24970-24971, 24980-24981, 24990-24991, 25000, 25002, 25010, 25012, 25020, 25022, 25030, 25032, 25040, 25042, 25050, 25052, 25060, 25062, 25070, 25072, 25080, 25082, 25090, 25092 | E109, E1065, E1010, E1069, E1011, E10641, E1029, E1021, E10311, E10319, E1036, E1037X1, E1037X1, E1037X2, E1037X3, E1037X9, E1039, E10311, E10319, E1036, E1039, E1040, E1051, E10618, E10620, E10621, E10622, E10628, E10630, E10638, E10649, E108, E108, E089, E099, E139, E0865, E0965, E0810, E0910, E1310, E0810, E0910, E0865, E0801, E0901, E1300, E0811, E08641, E0911, E09641, E1311, E13641, E0965, E0821, E0921, E08311, E08319, E0836, E0837X1, E0837X2, E0837X3, E0837X9, E0839, E09311, E09319, E0936, E0937X1, E0937X2, E0937X3, E0937X9, E0939, E1339, E0840, E0841, E0842, E0843, E0844, E0849, E08610, E0940, E0941, E0942, E0943, E0944, E0949, E09610, E1340, E1341, E1342, E1343, E1344, E1349, E0851, E0951, E1359, E08618, E08620, E08621, E08622, E08628, E08638, E0869, E09618, E09620, E09621, E09622, E09628, E09630, E09638, E09649, E0969, E13620, E13621, E13622, E13628, E13638, E13649, E1365, E1369, E088, E098, E138, E119, E1165, E1310, E1169, E1100, E1101, E1165, E11641, E1129, E1121, E11311, E11319, E1136, E1139, E1140, E1151, E11618, E11620, E11621, E11622, E11628, E11630, E11638, E11649, E118 |
| Infections (plus those below) | 0419, 042 | B9689, B20 |
| Hepatitis | 07022-07023, 07030-07033, 07041-07044, 07049, 07051-07054, 07059, 0706, 07070-07071 | B181, B180, B169, B1910, B161, B1711, B170, B172, B182, B178, B1710, B188, B189, B190, B1920, B1921 |
| Other viruses | 07951-07953, 07959, 0796, 07981-07983, 0785 | B9733, B9734, B9735, B333, B9739, B974, B334, B9721, B343, B259 |
| Pneumonia | 4800-4803, 4808-4809, 481, 4820-4822, 48230-48232, 48239-48242, 48249, 48281-48284, 48289, 4829-4831, 4838, 4841, 4843, 4845-4848, 485-486, 4870-4871, 4878, 48801-48802, 48809, 48811-48812, 48819, 48881-48882, 48889, 490, 4910-4911, 49120-49122, 4918-4919 | J120, J121, J122, J1281, J1289, J129, J13, J181, J150, J151, J14, J154, J153, J1520, J15211, J15212, J1529, J158, J155, J156, A481, J159, J157, J160, J168, B250, A3791, A221, B440, J17, J180, J189, J1100, J129, J101, J111, J112, J1181, J1189, J09X1, J09X2, J09X3, J09X9, J1008, J101, J40, J410, J411, J449, J441, J440, J418, J42 |
| Septicemia | 0380, 03810-03812, 03819, 0382-0383, 03840-03844, 03849, 0388 | A409, A412, A4101, A4102, A411, A403, A414, A4150, A413, A4151, A4152, A4153, A4159, A4189 |
| Intestinal infections | 00800-00804, 00809, 0081-0083, 00841-00847, 00849, 0085, 00861-00867, 00869, 0088, 0090-0093 | A044, A040, A041, A042, A043, A048, A045, A046, A047, A049, A080, A082, A0811, A0819, A0831, A0832, A0839, A088, A09 |
| Kidney/bladder infection | 59010-59011, 5902-5903, 59080-59081, 5909, 5950-5954, 5959, 5990 | N10, N151, N2884, N12, N16, N159, N3000, N3010, N3020, N3030, N3080, N3090, N390 |
| Tick borne | 0871, 0879-0880, 08881-08882, 08240-08241, 08249, 0828-0829 | A681, A689, A449, A6920, B600, A7740, A7741, A7749, A778, A799 |
| Growth factor use; HCPCS codes* | J1440-2, J1446-7, Q5101, J2505, J2820 | J1440-2, J1446-7, Q5101, J2505, J2820 |
| Acute autoimmune diseases | 136.1, 242.0, 245.2, 281.0, 283.0, 287.31, 364.3, 379.00, 374.53 | M35.2, E05.0, E06.3, D51.0, D59.1, D69.3, H20.9, H15.099, H02.739 |
| Chronic autoimmune diseases | 135, 255.41, 340, 357.0, 357.81, 358.0, 446.0, 446.4, 446.5, 555, 556, 571.42, 571.6, 579.0, 695.4, 696.0-696.1, 701.0, 709.01, 710, 710.0, 710.1, 710.2, 710.3, 710.4, 714.0-714.2, 714.8, 714.3, 720.0, 725 | D86.9, E27.1, G35, G61.0, G61.81, G70.0, M30.0, M31.30, M31.6, K50, K51, K75.4, K74.3, K90.0, L93.0, L40, L94.0, L80, M32.10, M34, M35.0, M33.1, M33.2, M05, M06, M08, M45, M35.3 |
| *Healthcare Common Procedure Coding System | | |
