# Supplementary material for: Risk Factors beyond Chemotherapy Exposure for Secondary Myeloid Neoplasms after Hematologic Cancers: A SEER-Based Study
Source: Cancer Res Commun. 2025 Dec 11;5(12):2149–56. doi: 10.1158/2767-9764.CRC-25-0340 (PMC12696405; doi:10.1158/2767-9764.CRC-25-0340)
Supplement: Supplemental Table S3 — Classification of Autoimmune Conditions Based on Immunological Status and Duration of Immunosuppression [file crc-25-0340_supplemental_table_s3_suppst3.docx]

| Condition | Deemed non-autoimmune | Deemed autoimmune with relatively longer duration of immunosuppression | Deemed autoimmune with shorter duration of immunosuppression |
| --- | --- | --- | --- |
| Autoimmune conditions (ICD-9 CM codes below) | 099.3; 242 (includes all causes of thyrotoxicosis: graves disease, toxic nodular goiter, toxic thyroid nodule, and lymphocytic thyroiditis); 281 (2810 Pernicious anemia already included) 357; 579 | 135.X; 245.2; 255.41; 340.X; 358; 446; 446.4; 446.5; 555.X; 556.X; 571.42; 571.6; 579.0; 695.4; 696.0-696.1; 701.0; 710; 710.1-710.4; 714; 720; 725.X | 136.1; 283; 287.31; 357.1; 364.3; 379; 374.53; 709.01; 725.X; 2810; 245.2 |
| ICD-9 CM code: 099.3 (ICD-10-CM M02.30 Reiter's disease), 135.X (ICD-10-CM D86.9 Sarcoidosis, unspecified), 136.1 (ICD-10-CM M35.2 Behçet's disease), 242 (Thyrotoxicosis with or without goiter), 245.2 (ICD-10-CM E06.3 Autoimmune thyroiditis), 255.41 (ICD-10-CM E27.1 Primary adrenocortical insufficiency), 281 (Other deficiency anemias, 281.0=Pernicious anemia), 283 (Acquired hemolytic anemias;283.0 Autoimmune hemolytic anemias), 287.31 (ICD-10-CM D69.3 Immune thrombocytopenic purpura), 340.X (ICD-10-CM G35 Multiple sclerosis), 357 (Inflammatory and toxic neuropathy: 357.1: Polyneuropathy due to collagen vascular disease) , 358 (Myoneural disorders; MG,Lambert Eaton Syndrome) , 364.3 (ICD-10-CM H20.9 Unspecified iridocyclitis) , 379 (Other disorders of eye including scleritis, episcleritis), 374.53 ( ICD-10-CM H02.739 Hypopigmentation of eyelid), 446 (Polyarteritis nodosa and allied conditions), 446.4 (ICD-10-CM M31.30 Wegener's granulomatosis without renal involvement), 446.5 (ICD-10-CM M31.6 Other giant cell arteritis), 555.X(Regional enteritis of SI, LI, unspecified sites) -556.X (UC), 571.42 (ICD-10-CM K75.4 Autoimmune hepatitis), 571.6 (Biliary cirrhosis (PBC SBC)), 579 (All causes of Intestinal malabsorptions, 579.0: Celiac disease) , 695.4 (ICD-10-CM L93.0 Discoid lupus erythematosus), 696.0-696.1 (Psoriasis and similar disorders), 701.0 (ICD-10-CM L94.0 Localized scleroderma ), 709.01 (Vitiligo), 710 (Diffuse diseases of connective tissue, 710.0 SLE ), 710.1-710.4 (710.1=Systemic sclerosis, 710.2:Sicca synd, 710.3:Dermatomyositis, 710.4:Polymyositis), 714 ( Rheumatoid arthritis and other inflammatory polyarthropathies), 720 (Ankylosing spondylitis and other inflammatory spondylopathies), 725.X (Polymyalgia rheumatica), 2810 (pernicious anemia- already included under 281) | | | |

**Supplemental Table S3**: Classification of Autoimmune Conditions Based on Immunological Status and Duration of Immunosuppression
