# Supplementary material for: Risk Factors beyond Chemotherapy Exposure for Secondary Myeloid Neoplasms after Hematologic Cancers: A SEER-Based Study
Source: Cancer Res Commun. 2025 Dec 11;5(12):2149–56. doi: 10.1158/2767-9764.CRC-25-0340 (PMC12696405; doi:10.1158/2767-9764.CRC-25-0340)
Supplement: Supplemental Table S4 — Risk of secondary MN after first primary DLBCL, FL, or MM diagnosed 2000-2011 using SEER-Medicare [file crc-25-0340_supplemental_table_s4_suppst4.docx]

|  | | | | | | | | | | | | | | | | | | | | | | | | | | | | | | |
| --- | --- | --- | --- | --- | --- | --- | --- | --- | --- | --- | --- | --- | --- | --- | --- | --- | --- | --- | --- | --- | --- | --- | --- | --- | --- | --- | --- | --- | --- | --- |
|  |  | DLBCL | | | | | | |  | | FL | | | | | | | |  | | MM | | | | | | | | | |
|  |  | **sMN** | **No sMN** | **HR** | **95% CI** | | **p-value** |  | | **sMN** | | **No sMN** | **HR** | **95% CI** | | **p-value** | |  | | **sMN** | | **No sMN** | | **HR** | | **95% CI** | | | **p-value** | |
|  |  | n=157 | n=14,427 |  |  |  |  |  | | n=117 | | n=9,998 |  |  |  |  |  |  | | n=98 | | n=14,804 | |  |  |  |  |  |  |  |
| **Age at first primary cancer** | |  |  |  |  |  | <0.0001 |  | |  | |  |  |  |  | | <0.0001 | |  | |  | |  | |  | |  |  | 0.7 | |
|  | <70 years | 47 | 3393 | ref |  |  |  |  | | 36 | | 2690 | ref |  |  | |  | |  | | 30 | | 3619 | | ref | |  |  |  | |
|  | 70-<75 years | 47 | 4147 | 1.37 | (0.91 | , 2.06) |  |  | | 31 | | 2968 | 1.40 | (0.86 | , 2.27) | |  | |  | | 30 | | 4408 | | 1.08 | | (0.64 | , 1.82) |  | |
|  | ≥75 years | 63 | 6887 | 2.65 | (1.71 | , 4.13) |  |  | | 50 | | 4340 | 3.66 | (2.18 | , 6.16) | |  | |  | | 38 | | 6777 | | 1.24 | | (0.71 | , 2.17) |  | |
| **Initial chemotherapy/G-CSF** | | |  |  |  |  | <0.0001 |  | |  | |  |  |  |  | | <0.0001 | |  | |  | |  | |  | |  |  | <0.0001 | |
|  | no chemotherapy or G-CSF | 47 | 7368 | ref |  |  |  |  | | 38 | | 6076 | ref |  |  | |  | |  | | 41 | | 9486 | | ref | |  |  |  | |
|  | chemotherapy or G-CSF | 35 | 4463 | 1.20 | (0.78 | , 1.84) |  |  | | 26 | | 2766 | 1.50 | (0.92 | , 2.44) | |  | |  | | 32 | | 4656 | | 1.70 | | (1.07 | , 2.72) | |  |
|  | chemotherapy and G-CSF | 75 | 2596 | 4.56 | (3.23 | , 6.43) |  |  | | 53 | | 1156 | 6.79 | (4.48 | , 10.28) | |  | |  | | 25 | | 662 | | 8.77 | | (5.21 | , 14.79) | |  |
| **Cardiovascular disease** | |  |  |  |  |  | 0.4 |  | |  | |  |  |  |  | | 0.1 | |  | |  | |  | |  | |  |  | 0.8 | |
|  | no cardiovascular disease | 31 | 2350 | ref |  |  |  |  | | 25 | | 1770 | ref |  |  | |  | |  | | 17 | | 2405 | | ref | |  |  |  | |
|  | cardiovascular disease | 126 | 12077 | 0.83 | (0.56 | , 1.24) |  |  | | 92 | | 8228 | 0.71 | (0.46 | , 1.09) | |  | |  | | 81 | | 12399 | | 0.94 | | (0.53 | , 1.67) |  | |
| **Acute autoimmune conditions** | | |  |  |  |  | 0.4 |  | |  | |  |  |  |  | | 0.04 | |  | |  | |  | |  | |  |  | 0.3 | |
|  | no autoimmune conditions | 143 | 13305 | ref |  |  |  |  | | 103 | | 9295 | ref |  |  | |  | |  | | 87 | | 13679 | | ref | |  |  |  | |
|  | autoimmune conditions | 14 | 1122 | 1.25 | (0.71 | , 2.20) |  |  | | 14 | | 703 | 1.85 | (1.04 | , 3.29) | |  | |  | | 11 | | 1125 | | 1.42 | | (0.75 | , 2.69) |  | |
| **Chronic autoimmune conditions** | | |  |  |  |  | 0.02 |  | |  | |  |  |  |  | | 0.2 | |  | |  | |  | |  | |  |  | 0.8 | |
|  | no autoimmune conditions | 121 | 12025 | ref |  |  |  |  | | 96 | | 8510 | ref |  |  | |  | |  | | 83 | | 12644 | | ref | |  |  |  | |
|  | autoimmune conditions | 36 | 2402 | 1.60 | (1.09 | , 2.37) |  |  | | 21 | | 1488 | 1.41 | (0.85 | , 2.32) | |  | |  | | 15 | | 2160 | | 1.07 | | (0.62 | , 1.84) |  | |
| **Infection** | |  |  |  |  |  | 0.01 |  | |  | |  |  |  |  | | 0.8 | |  | |  | |  | |  | |  |  | 0.02 | |
|  | no infection | 63 | 5078 | ref |  |  |  |  | | 40 | | 3843 | ref |  |  | |  | |  | | 34 | | 4299 | | ref | |  |  |  | |
|  | infection | 94 | 9349 | 0.67 | (0.49 | , 0.92) |  |  | | 77 | | 6155 | 0.95 | (0.66 | , 1.39) | |  | |  | | 64 | | 10505 | | 0.60 | | (0.39 | , 0.93) |  | |
| *Models controlled for months of Medicare coverage.  Abbreviations: CI – confidence interval, DLBCL – diffuse large B-cell lymphoma, FL – follicular lymphoma, G-CSF - granulocyte colony-stimulating factor, HR – hazard ratio, MM – multiple myeloma, sMN – secondary myeloid neoplasm. | | | | | | | | | | | | | | | | | | | | | | | | | | | | | | |

**Supplemental Table S4**: Risk of secondary MN after first primary DLBCL, FL, or MM diagnosed 2000-2011 using SEER-Medicare
