# Supplementary material for: Risk Factors beyond Chemotherapy Exposure for Secondary Myeloid Neoplasms after Hematologic Cancers: A SEER-Based Study
Source: Cancer Res Commun. 2025 Dec 11;5(12):2149–56. doi: 10.1158/2767-9764.CRC-25-0340 (PMC12696405; doi:10.1158/2767-9764.CRC-25-0340)
Supplement: Supplemental Table S5 — Risk of sMN after first primary DLBCL diagnosed 2000-2011 using SEER-Medicare [file crc-25-0340_supplemental_table_s5_suppst5.docx]

|  | | | | | | | |
| --- | --- | --- | --- | --- | --- | --- | --- |
|  |  | **sMN** | **No sMN** | **HR** | **95% CI** | | **p-value** |
|  |  | n=157 | n=14,427 |  |  |  |  |
| **Age at first primary cancer** | |  |  |  |  |  | <0.0001 |
|  | <70 years | 47 | 3393 | ref |  |  |  |
|  | 70-<75 years | 47 | 4147 | 1.37 | (0.91 | , 2.05) |  |
|  | ≥75 years | 63 | 6887 | 2.64 | (1.70 | , 4.12) |  |
| **Initial chemotherapy/G-CSF** | |  |  |  |  |  | <0.0001 |
|  | no chemotherapy or G-CSF | 47 | 7368 | ref |  |  |  |
|  | chemotherapy or G-CSF | 35 | 4463 | 1.17 | (0.76, | 1.81) |  |
|  | chemotherapy and G-CSF | 75 | 2596 | 4.45 | (3.12 | , 6.36) |  |
| **Chronic autoimmune conditions** | | |  |  |  |  | 0.02 |
|  | no autoimmune conditions | 121 | 12025 | ref |  |  |  |
|  | autoimmune conditions | 36 | 2402 | 1.60 | (1.10 | , 2.34) |  |
| **Infection** | |  |  |  |  |  | 0.006 |
|  | no infection | 63 | 5078 | ref |  |  |  |
|  | infection | 94 | 9349 | 0.65 | (0.48 | , 0.89) |  |
| *Models controlled for months of Medicare coverage. | | | | | | | |
| Abbreviations: CI – confidence interval, DLBCL – diffuse large B-cell lymphoma, G-CSF - granulocyte colony-stimulating factor, HR – hazard ratio, sMN – secondary myeloid neoplasm. | | | | | | | |

**Supplemental Table S5**: Risk of sMN after first primary DLBCL diagnosed 2000-2011 using SEER-Medicare
