# Supplementary material for: Risk Factors beyond Chemotherapy Exposure for Secondary Myeloid Neoplasms after Hematologic Cancers: A SEER-Based Study
Source: Cancer Res Commun. 2025 Dec 11;5(12):2149–56. doi: 10.1158/2767-9764.CRC-25-0340 (PMC12696405; doi:10.1158/2767-9764.CRC-25-0340)
Supplement: Supplemental Table S6 — Risk of sMN after first primary FL diagnosed 2000-2011 using SEER-Medicare [file crc-25-0340_supplemental_table_s6_suppst6.docx]

|  | | | | | | | |
| --- | --- | --- | --- | --- | --- | --- | --- |
|  |  | **sMN** | **No sMN** | **HR** | **95% CI** | | **p-value** |
|  |  | n=117 | n=9,998 |  |  |  |  |
| **Age at first primary cancer** | |  |  |  |  |  | <0.0001 |
|  | <70 years | 36 | 2690 | ref |  |  |  |
|  | 70-<75 years | 31 | 2968 | 1.36 | (0.84 | , 2.21) |  |
|  | ≥75 years | 50 | 4340 | 3.33 | (1.96 | , 5.63) |  |
| **Initial chemotherapy/G-CSF** | |  |  |  |  |  | <0.0001 |
|  | no chemotherapy or G-CSF | 38 | 6076 | ref |  |  |  |
|  | chemotherapy or G-CSF | 26 | 2766 | 1.42 | (0.86 | , 2.34) |  |
|  | chemotherapy and G-CSF | 53 | 1156 | 6.32 | (4.11 | , 9.72) |  |
| **Acute autoimmune conditions** | | |  |  |  |  | 0.03 |
|  | no autoimmune conditions | 103 | 9295 | ref |  |  |  |
|  | autoimmune conditions | 14 | 703 | 1.86 | (1.05 | , 3.29) |  |
| **Year of first primary cancer** | |  |  |  |  |  | 0.04 |
|  | 2000-2003 | 55 | 3142 | ref |  |  |  |
|  | 2004-2011 | 34 | 3436 | 0.68 | (0.47 | , 0.99) |  |
| *Models controlled for months of Medicare coverage. | | | | | | | |
| Abbreviations: CI – confidence interval, FL – follicular lymphoma, G-CSF - granulocyte colony-stimulating factor, HR – hazard ratio, sMN – secondary myeloid neoplasm. | | | | | | | |

**Supplemental Table S6**: Risk of sMN after first primary FL diagnosed 2000-2011 using SEER-Medicare
