# Supplementary material for: Risk Factors beyond Chemotherapy Exposure for Secondary Myeloid Neoplasms after Hematologic Cancers: A SEER-Based Study
Source: Cancer Res Commun. 2025 Dec 11;5(12):2149–56. doi: 10.1158/2767-9764.CRC-25-0340 (PMC12696405; doi:10.1158/2767-9764.CRC-25-0340)
Supplement: Supplemental Table S7 — Risk of sMN after first primary MM diagnosed 2000-2011 using SEER-Medicare [file crc-25-0340_supplemental_table_s7_suppst7.docx]

|  | | | | | | | |
| --- | --- | --- | --- | --- | --- | --- | --- |
|  |  | MM | | | | | |
|  |  | **sMN** | **No sMN** | **HR** | **95% CI** | | **p-value** |
|  |  | n=98 | n=14,804 |  |  |  |  |
| **Initial chemotherapy/G-CSF** | |  |  |  |  |  | <0.0001 |
|  | no chemotherapy or G-CSF | 41 | 9486 | ref |  |  |  |
|  | chemotherapy or G-CSF | 32 | 4656 | 1.73 | (1.10 | , 2.72) |  |
|  | chemotherapy and G-CSF | 25 | 662 | 9.43 | (5.75 | , 15.47) |  |
| **Infection** | |  |  |  |  |  | 0.01 |
|  | no infection | 34 | 4299 | ref |  |  |  |
|  | infection | 64 | 10505 | 0.59 | (0.40 | , 0.89) |  |
| Abbreviations: CI – confidence interval, G-CSF - granulocyte colony-stimulating factor, HR – hazard ratio, MM – multiple myeloma, sMN – secondary myeloid neoplasm. | | | | | | | |

**Supplemental Table 7**: Risk of sMN after first primary MM diagnosed 2000-2011 using SEER-Medicare
